# Impact of the COVID-19 pandemic on risk of burn-out syndrome and recovery need among secondary school teachers in Flanders: A prospective study

**DOI:** 10.3389/fpubh.2022.1046435

**Published:** 2022-12-12

**Authors:** Hannah De Laet, Yanni Verhavert, Kristine De Martelaer, Evert Zinzen, Tom Deliens, Elke Van Hoof

**Affiliations:** ^1^Department of Psychology, Vrije Universiteit Brussel, Brussels, Belgium; ^2^Department of Movement and Sport Sciences, Vrije Universiteit Brussel, Brussels, Belgium

**Keywords:** teaching staff, quarantine, lockdown, emotional exhaustion, depersonalization, personal accomplishment, mental health, longitudinal

## Abstract

**Background:**

Due to the COVID-19 pandemic, schools were closed, teachers had to teach from home and after a while, they had to return to the classroom while the pandemic was still on-going. Even before the pandemic, teachers were already more at risk for burn-out syndrome compared to the general population. Furthermore, not much research pertaining to this population has been carried out during the pandemic and so the impact of the pandemic on teachers' risk of burn-out syndrome and recovery need remains unclear. The aim of the current study was to fill this knowledge gap and map out the impact on risk of burn-out syndrome and recovery need at different time points during the pandemic.

**Methods and findings:**

At baseline, 2,167 secondary school teachers in Flanders were included in this prospective study. Questionnaire data were obtained at ten different time points between September 2019 and August 2021. To assess risk of burn-out syndrome and its dimensions, the Utrecht Burn-out Scale for Teachers was administered. Need for recovery was assessed using questions adopted from the Short Inventory to Monitor Psychosocial Hazards. The results revealed an initial positive effect of the first lockdown (Mar/Apr 2020) with a decrease in risk of burn-out syndrome [Odds ratio (OR) Jan/Feb 2020–Mar/Apr 2020 = 0.33, *p* < 0.001], emotional exhaustion (EMM Jan/Feb 2020–Mar/Apr 2020 = −0.51, *p* < 0.001), depersonalization (EMM Jan/Feb 2020–Mar/Apr 2020 = −0.13, *p* < 0.001) and recovery need [Estimated marginal mean (EMM) Jan/Feb 2020–Mar/Apr 2020 = −0.79, *p* < 0.001]. No significant effect on personal accomplishment was found (*p* = 0.410). However, as the pandemic went on, higher risk of burn-out syndrome, emotional exhaustion, depersonalization and recovery need, and lower personal accomplishment were observed.

**Conclusions:**

Despite the initial positive impact on risk of burn-out syndrome, its dimensions and recovery need, a negative long-term impact of the COVID-19 pandemic became visible. This study highlights once again the importance for interventions to reduce teachers' risk of burn-out syndrome, especially in such difficult times as a pandemic.

## Introduction

Besides the severe medical impact of COVID-19, the far-reaching measures taken in many countries to contain the virus also dramatically impacted our daily lives (1, 2), which in turn influenced our mental health (3, 4). Lockdowns were installed, which resulted in closure of schools, bars and restaurants and cancellation of all social and cultural events (5, 6). During the first weeks of the lockdown in Belgium (i.e., 16th of March 2020–20th of April 2020), schools were closed and no alternative way of teaching was organized. Later on, schools started online teaching, resulting in considerable implications for the teaching professionals. Teachers had to adapt to digitalized long-distance learning and teaching, and giving assignments through online platforms (5, 7, 8). After the first lockdown (i.e., March–April 2020), they moved to hybrid learning (i.e., a mixture of digital and face-to-face teaching), and thus they had to figure out how to combine online and face-to-face teaching, or teaching from home one week and in person the next week (9). Returning to the classroom also meant that teachers had to put themselves and their families at risk for infection while the pandemic was still ongoing (6, 10, 11).

During non-COVID times, it was found that teachers are particularly at risk for occupational stress that could lead to depression and burn-out syndrome (12–15). Burn-out syndrome rates in teachers are also significantly higher compared to other professions (15, 16). A study in Finland found 12% of teachers (across education levels) to experience stress and burn-out syndrome while this was only 8% in other professions (16). Moreover, in Belgium it was found that 21% of teachers (across education levels) reported burn-out symptoms compared to 13% in the general population (17). Higher burn-out syndrome rates also result in lower teacher wellbeing and higher absenteeism and turnover rates (15). A report about sick leave in Flemish secondary school teachers reported an average of 16.4 days of sick leave in 2019, of which 42.8% were due to psychosocial diseases such as burn-out syndrome (18). Different factors, such as perceived self-efficacy, job satisfaction and high social support, have been related to lower burn-out syndrome rates in teachers (19–21). Factors such as role ambiguity and high job demand on the other hand were found to be related to higher burn-out syndrome rates (19–21). Given the extra demands on teachers during the pandemic, they might be more at risk of burn-out syndrome compared to other professions.

Burn-out syndrome can be defined as a “prolonged response to chronic emotional and interpersonal stressors on the job, determined by three dimensions: emotional exhaustion, cynicism or depersonalization, and professional (in)efficacy or personal accomplishment” (22). Burn-out syndrome consists of three dimensions, namely, emotional exhaustion, depersonalization, and personal accomplishment (22). Emotional exhaustion refers to a lack of energy and depletion of an individual's emotional resources due to work (22). Depersonalization is defined as a mental distance from work or an impersonal attitude toward students and colleagues (22). Personal accomplishment refers to feeling competent in the work being carried out and the contact with others (22). Besides burn-out syndrome, recovery need should be equally considered, as before a burn-out syndrome is present, individuals are often not able to recover from stress anymore (23). After a while of having a high need for recovery while not being able to recover, the stress system adapts itself resulting in chronically high stress levels and, later on burn-out syndrome (23). As long as people are able to recover from the (stressful) day they have had, the risk for burn-out syndrome remains low but when they are not able to recover, this risk increases (23). Need for recovery and burn-out syndrome are thus closely related (23).

During the COVID-19-pandemic, a decline in wellbeing and an increase in mental health problems were observed in the general population (3, 4). During periods of less strict measures, wellbeing increased, and the number of mental health problems decreased (24, 25). A recent report by the World Health Organization found an increase of 25% in anxiety and depression during the pandemic (26). However, as soon as measures became stricter again, deteriorations in mental health were visible (24, 25). Even though measures and their strictness differed among countries, comparable results regarding the impact of the COVID-19 pandemic on mental health were found across borders (4, 24, 25).

In comparison with other at-risk groups, such as healthcare workers and youngsters, research on mental health of teachers during the pandemic is scarce. Previous studies focusing on mental health in teachers during the pandemic mainly used a cross-sectional design (10, 27–31), and thus precluding comparison with data collected prior to the pandemic. In general, these studies found increased levels of depression, anxiety and high levels of stress during the pandemic (27, 31–37). However, a few studies also found a positive effect of the pandemic on teachers' mental health. This was mainly the case in the first few months of lockdown (11, 28, 38). Regarding burn-out syndrome levels in teachers during the pandemic, only a few studies, showing contradicting results, were found. A study by Pereira et al. (39) found teachers to have overall low levels of burn-out syndrome during the pandemic, whereas other studies showed increased levels of burn-out syndrome (40, 41) and its dimensions (35, 40, 42, 43). However, these studies were not able to compare the levels of burn-out syndrome during the pandemic to the levels of burn-out syndrome before. Hence, the effect of the pandemic on burn-out syndrome among teachers remains unclear, nor is there any evidence on recovery need, which may partially mediate the relationship between work stress and burn-out syndrome (23, 44).

This study presents unique natural experiment data on how the COVID-19 pandemic impacted risk of burn-out syndrome and its dimensions, and recovery need over a two-year timespan in Flemish secondary school teachers. The research objective of this study is to map out the changes in risk of burn-out syndrome and recovery need in secondary school teachers during the COVID-19 pandemic.

## Methods

### Participants

Flemish secondary school teachers were recruited in August and September 2019 using a non-probability cluster sampling approach. We contacted all secondary schools in Flanders (Belgium) through e-mail and telephone and invited them to participate. The Flemish Department of Education (*Vlaams Departement Onderwijs*), as well as all education networks (i.e., Flemish community schools, subsidized public schools, subsidized free schools) were involved in the recruitment and were asked to promote the study among all school principals and by posting advertisements on their social media. Furthermore, a convenient selection of schools in Flanders were visited to promote our study face-to-face. Schools that were willing to participate were asked to share an e-mail with a link to the online questionnaire with their teaching staff. The link to the online questionnaire was also spread through social media (e.g., Facebook and Twitter). Teachers not teaching in secondary education and teachers being in sick leave due to illness were excluded from the final sample.

### Design and procedure

**Figure 1:**
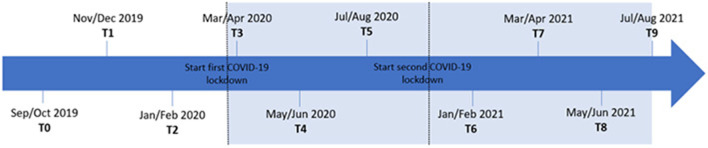
Timing of the measurements.

This prospective cohort study is part of another longitudinal study (investigating the association between risk of burn-out syndrome and energy balance related behavior), including six time points throughout the 2019–2020 school year [i.e., Sep/Oct (T0), Nov/Dec (T1), Jan/Feb (T2), Mar/Apr (T3), May/Jun (T4), and Jul/Aug (T5)]. For the purpose of the present study (i.e., measuring the (long-term) impact of COVID-19 on secondary school teachers' risk of burn-out syndrome and recovery need), four extra measurements were conducted during the 2020–2021 school year [i.e., Jan/Feb (T6), Mar/Apr (T7), May/Jun (T8), and Jul/Aug (T9)]. Measurements were not conducted in Sep/Oct and Nov/Dec of the 2020–2021 school year, as it was decided on an *ad hoc* basis to conduct additional measurements when it became clear that COVID-19 had not yet come to an end. The measurements T0, T1, and T2 of the school year 2019–2020 can be defined as pre-pandemic measurements, while all other measurements took place during the COVID-19 pandemic. The timeline of the measurements is displayed in Figure 1. At all time points, teachers were asked to complete an online questionnaire, including questions regarding burn-out syndrome, recovery need, socio-demographics and work-related factors. During each measurement period of two weeks, three reminders were sent to the non-responders, each on the 4th, 8th, and 11th day after activation of the online questionnaire.

### Measures

#### Risk of burn-out syndrome

Risk of burn-out syndrome was assessed using the Dutch version of the validated Maslach Burn-out Inventory (MBI) (45): Utrechtse Burn-out Schaal voor Leerkrachten (UBOS-L; Utrecht Burn-out Scale for Teachers) (46). The UBOS-L is especially developed for teachers, administrators and other staff members working in educational settings and assesses the three dimensions of burn-out syndrome (i.e., emotional exhaustion, depersonalization, and personal accomplishment). The questionnaire consists of 22 items (8 items relate to emotional exhaustion, 7 items to depersonalization and 7 items to personal accomplishment) presented on a 7-point Likert scale ranging from 0 (never) to 6 (every day). An average score of each dimension was calculated by dividing each scale score by the number of items. The three dimensions' scores were then combined to calculate risk of burn-out syndrome. Individuals scoring high on emotional exhaustion (i.e., > 2.5) and low on personal accomplishment (i.e., < 3.56), or high on emotional exhaustion (i.e., > 2.5) and high on depersonalization [i.e., > 1.43 (males) and > 2.00 (females)], are considered at risk for burn-out syndrome based on the UBOS-L norms (46). This validated questionnaire showed a good internal consistency (Emotional exhaustion: Cronbach's alpha = 0.91; Depersonalization: Cronbach's alpha = 0.73; Personal accomplishment: Cronbach's alpha = 0.85) and a good test-retest reliability (Emotional exhaustion: Pearson's *r* = 0.81; Depersonalization: Pearson's *r* = 0.65; Personal accomplishment: Pearson's *r* = 0.72).

#### Recovery need

Recovery need was assessed by using the validated Short Inventory to Monitor Psychosocial Hazards (SIMPH) (47). Only the “recovery need” part of this questionnaire, including 5 yes (= 1)/no (= 0) questions, was used. A total score on 5 was calculated. Participants having a score ≥ 3/5 were classified in the category “high need for recovery”. The part “recovery need” of this questionnaire showed a good reliability (Cronbach's alpha = 0.78).

#### Socio-demographics and work-related information

Socio-demographics include sex, age, highest diploma (i.e., secondary school degree, post-secondary school degree or certificate, Bachelor's degree, Master's degree, PhD degree), marital status (i.e., single, married, unmarried, living together with partner, divorced, widowed), having children (yes/no) and ethnicity (i.e., White–European, White–other, North-African, Afro-American, Indian, Middle-Eastern, South-Asian, Southeast-Asian, other). Work-related factors include grade (1st, 2nd, and 3rd), school type (i.e., general secondary education, technical secondary education, art secondary education, vocational secondary education), school, education network (i.e., Flemish community schools, subsidized public schools, subsidized free schools) and total teaching hours per week.

### Ethics statement

This study was conducted in accordance with the Helsinki Declaration and its later amendments. Written informed consent was obtained from all participants prior to study enrolment. The study protocol was approved by the Medical Ethics Committee of the University Hospital (UZ Brussel, Brussels, Belgium; B.U.N. 143201940533).

### Statistical analyses

All data were analyzed using R [R core Team, (48); R Studio version 3.6.2]. *P*-values <0.05 were considered statistically significant. Representativeness of the sample at baseline (T0) was assessed by conducting two proportions z-tests. Regarding the outcomes emotional exhaustion, depersonalization, personal accomplishment, risk of burn-out syndrome and recovery need, drop-out analyses between each consecutive time point and between each time point and baseline (T0) were conducted to assess possible differences between the drop-out and retention group, and thus possible selection bias of the retention group. Additionally, drop-out analyses regarding sex and age (which are non-fluctuating variables over time) were performed between each time point and baseline (T0).

Preliminary analyses checked if a multilevel model was advised (repeated measures clustered within participants, participants clustered within grades or school types or schools or education networks) by inspecting the amount of variance explained by each cluster. If necessary, one (or more) levels were dropped. For the continuous outcomes, general linear mixed models were applied using the R package lme4 (49). Regarding the categorical outcome, generalized linear mixed models (i.e., the Binominal model) were applied, also using the R package lme4 (49). Models were built bottom-up starting with the intercept only model, adding first level predictors and second level predictors. Confounders (i.e., age, sex, and teaching hours per week) that did not statistically impact the outcome were temporarily removed from the model and an ANOVA comparing the original model to the reduced model was performed. Furthermore, to decide upon which confounders had to be taken into account, Akaike Information Criterion (AIC) values were compared. When no statistical difference was found between the full and reduced model and the AIC did not improve, the confounder was removed from the model. The model selection procedure of each outcome is explained in Appendix S1. To compare each time point to the subsequent time point, the contrasts were set to the successive difference of the treatment means.

## Results

**Figure 2:**
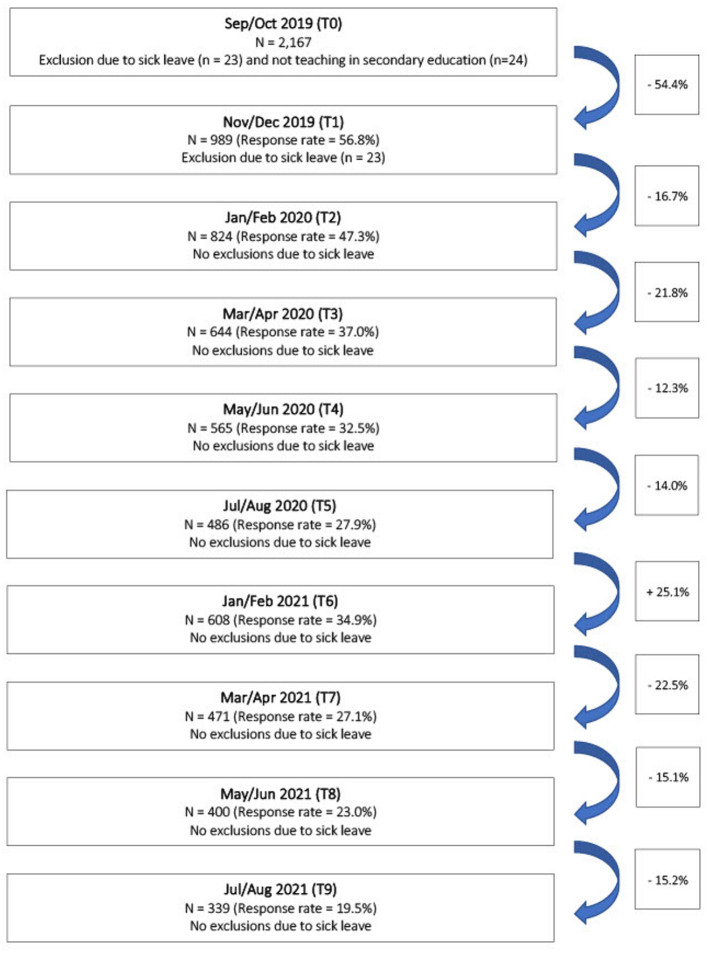
Flow chart with drop-out rates across all time points.

Two thousand one hundred ninety-seven secondary school teachers filled in the first questionnaire at the start of the larger study (T0; Sep/Oct, 2019), of which 1,741 provided their e-mail address and thus consented to be recontacted for each following time point. Of the initial 2,197 participants, 2,167 remained after exclusion (i.e., sick leave (*n* = 23) and not working in secondary education (*n* = 24). At the final time point (T9; Jul/Aug, 2021), three hundred thirty-nine participants completed the last questionnaire, which corresponds to a total drop-out rate of 84.4% across the complete measurement period. More detailed information regarding drop-out rates and the number of excluded participants across all time points can be found in Figure 2.

### Sample characteristics

At baseline (T0), the sample included 2,167 participants consisting of 77.6% females and having a mean age of 42.0 ± 10.2 years. At the start of the school year, about 1/5 of secondary school teachers reported to be at risk for burn-out syndrome, while more than half reported to have a high need for recovery. More detailed information regarding sample characteristics can be found in Table 1.

**Table 1:** Baseline sample characteristics (T0; *n* = 2,167).

	**Mean ±SD; %**
Sex (% females)	77.6
Age (years)	42.0 ± 10.2
**Diploma (%)**	
Secondary school degree	1.5
Post-secondary school degree	1.8
Bachelor's degree	59.2
Master's degree	37.6
PhD degree	0.9
Having an extra job (%)	13.8
**Marital status (%)**	
Single	13.1
Married	51.7
Unmarried	5.0
Living together with partner	23.9
Divorced	5.7
Widowed	0.6
Having children (%)	72.3
**Ethnicity (%)**	
White, European	99.0
White, other	0.1
North-African	0.3
Middle Eastern	0.4
Southeast Asian	0.1
Mixed	0.1
**Education network (%)**	
Flemish community schools	51.1
Subsidized free schools	45.0
Subsidized public schools	3.5
Mixed	0.4
Teaching hours per week (hours/week)	18.9 ± 5.0
Teaching experience (years)	14.8 ± 9.4
Risk of burn-out syndrome (%)	20.8
Need for recovery (%)	55.7

*SD*, standard deviation.

### Representativeness of the baseline sample

The baseline sample was not representative for sex (i.e., sample vs. population: males: 22.4 vs. 35.1%, females: 77.6 vs. 64.9%; *p* < 0.001), age groups 20–29 (i.e., sample vs. population: 12.2 vs. 14.8%; *p* < 0.001) and 30–39 (i.e., sample vs. population: 32.5 vs. 29.4%; *p* = 0.001) and education network (i.e., sample vs. population: Flemish community schools: 51.3 vs. 22.5%, subsidized free schools: 45.2 vs. 68.0%, subsidized public schools: 3.5 vs. 9.4%; *p* < 0.001). Details regarding the representativeness of the sample can be found in Appendix S2.

### Drop-out analyses

Drop-out analyses between each consecutive time point showed a significant difference in recovery need between Jan/Feb 2020 (T2) and Nov/Dec 2019 (T1) between the retention and drop-out group. On average, drop-outs showed a higher recovery need (2.95 ± 1.67) than those who remained in the study (2.75 ± 1.74; *p* = 0.0448). Between other consecutive time points, no significant differences were found between both the retention and drop-out groups for any of the outcome measures, namely, risk of burn-out syndrome, emotional exhaustion, depersonalization, personal accomplishment and recovery need. More detailed information can be found in Table A2 in Appendix S2.

Drop-out analyses between each time point and baseline (T0) showed significant differences for age at all time points (all *p*-values < 0.001), with the retention group being slightly older (around 2–4 years, depending on the time point) than the drop-out group. More detailed information can be found in Table A3 in Appendix S2. From T6 onwards, the proportion of females was more or less 6% higher in the retention group compared to the drop-out group (all *p*-values < 0.05). At T1-5, no significant sex-differences between the retention and the drop-out group were observed. More detailed information can be found in Table A3 in Appendix S2.

### Changes in risk of burn-out syndrome, burn-out syndrome dimensions and recovery need over time

Percentages of risk of burn-out syndrome and recovery need ranged from 20.8 to 30.8 and 34.0 to 61.4%, respectively, across all time points. More detailed information can be found in Appendix S3.

The estimated marginal means/odds ratios and standard errors as well as the estimates of the predictors for all outcomes across all time points can be found in Appendix S4. Three levels (repeated measures clustered within participants clustered within schools) were included in the models. The models including grade, school type and education network in which the respondent was teaching, showed that hardly any variance was explained by these levels. Therefore, these levels were not included in the final models, assuming that the time effect is invariant across grades, school types and/or education networks. All models include random intercepts for the participants.

**Figure 3:**
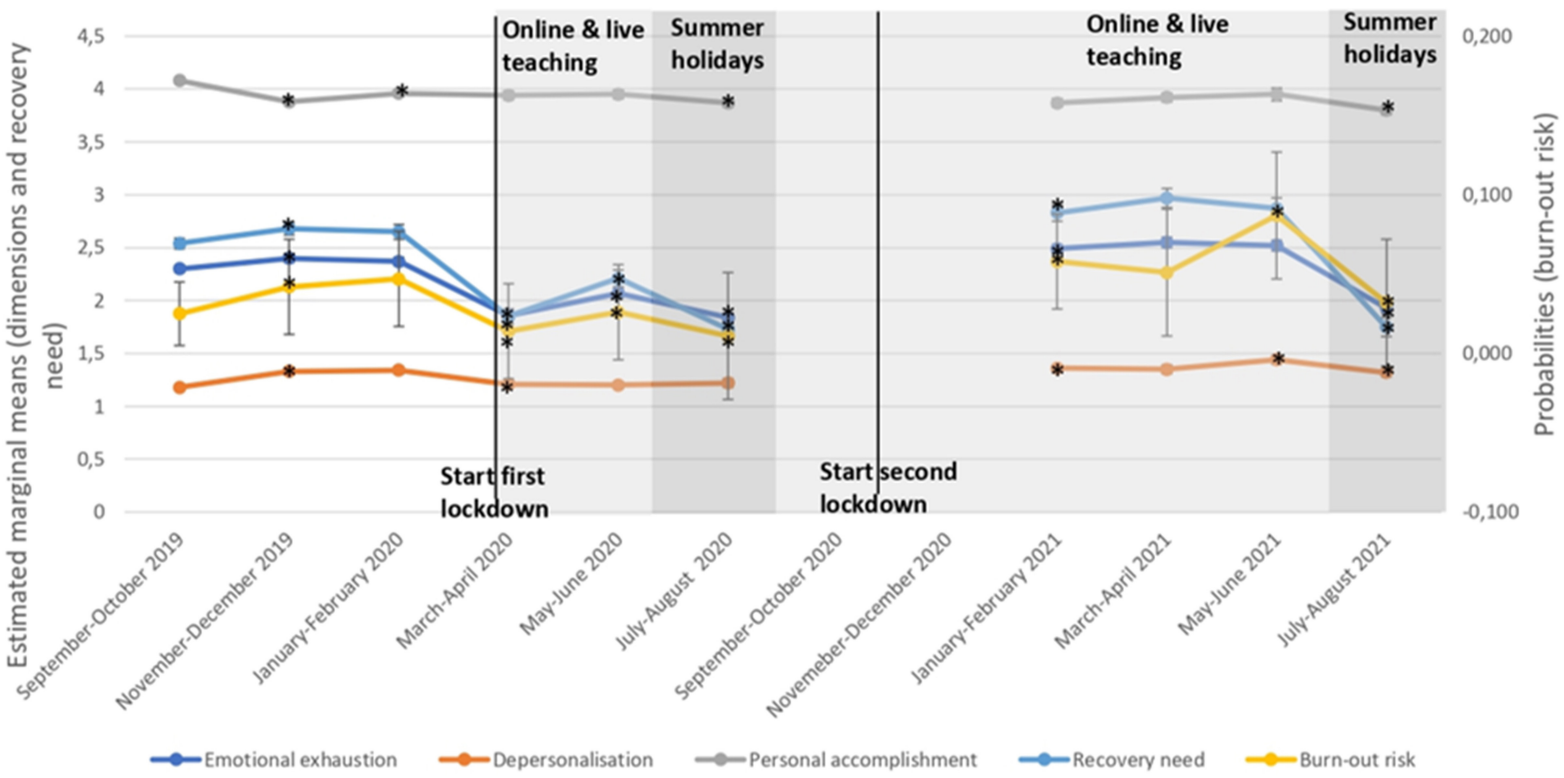
Changes in burnout risk, burnout dimensions and recovery need over time. *Time point significantly different (p < 0.05) from previous time point.

From Sep/Oct 2019 to Nov/Dec 2019, risk of burn-out syndrome (OR = 1.83, *p* < 0.001), emotional exhaustion (Estimated marginal mean (EMM) = 0.10, *p* = 0.001), depersonalization (EMM = 0.15, *p* < 0.001) and recovery need (EMM = 0.14, *p* = 0.010) significantly increased, whereas personal accomplishment significantly decreased (EMM = −0.15, *p* < 0.001). From Nov/Dec 2019 to Jan/Feb 2020, only a significant increase in personal accomplishment was found (EMM = 0.08, *p* = 0.009). At the time of the first lockdown, so from Jan/Feb 2020 to Mar/Apr (2020), risk of burn-out syndrome (OR = 0.33, *p* < 0.001), emotional exhaustion (EMM = −0.51, *p* < 0.001), depersonalization (EMM = −0.13, *p* < 0.001) and recovery need (EMM = −0.79, *p* < 0.001) significantly decreased. From Mar/Apr 2020 to May/Jun 2020, risk of burn-out syndrome (OR = 2.61, *p* < 0.001), emotional exhaustion (EMM = 0.21, *p* < 0.001) and recovery need (EMM = 0.36, *p* < 0.001) significantly increased. From May/Jun 2020 to Jul/Aug 2020 (i.e., summer holidays 2020), risk of burn-out syndrome (OR = 0.32, *p* < 0.001), emotional exhaustion (EMM = −0.23, *p* < 0.001), personal accomplishment (EMM = −0.08, *p* = 0.048) and recovery need (EMM = −0.48, *p* < 0.001) significantly decreased. After the summer holidays, so from Jul/Aug 2020 to Jan/Feb 2021 (including the second lockdown), significant increases in risk of burn-out syndrome (OR = 5.30, *p* < 0.001), emotional exhaustion (EMM = 0.65, *p* < 0.001), depersonalization (EMM = 0.13, *p* < 0.001) and recovery need (EMM = 1.10, *p* < 0.001) were found. No significant differences were found from Jan/Feb 2021 to Mar/Apr 2021. From Mar/Apr 2021 to May/Jun 2021, only risk of burn-out syndrome (OR = 1.85, *p* = 0.036) and depersonalization increased significantly (EMM = 0.09, *p* = 0.032). Lastly, significant decreases were found from May/Jun 2021 to Jul/Aug 2021 (i.e., summer holidays 2021) for risk of burn-out syndrome (OR = 0.28, *p* < 0.001), emotional exhaustion (EMM = −0.60, *p* < 0.001), depersonalization (EMM = −0.12, *p* = 0.005), personal accomplishment (EMM = −0.16, *p* = 0.002) and recovery need (EMM = −1.12, *p* < 0.001). All changes over time in risk of burn-out syndrome, its dimensions and recovery need are displayed in Figure 3.

## Discussion

This prospective study investigated changes of risk of burn-out syndrome, its dimensions and recovery need in secondary school teachers prior to and during the COVID-19 pandemic including ten different time points. Across all time points, we observed a high percentage of teachers being at risk for burn-out syndrome (ranging between 20.8 and 30.8%) and having a high recovery need (ranging between 34.0 and 61.4%). An initial positive effect of the pandemic was found with a decrease in risk of burn-out syndrome and recovery need. However, risk of burn-out syndrome and recovery need showed increased values as the pandemic went on.

Overall, about 1/5–1/3 of teachers in the current study were at risk for burn-out syndrome. This is in line with a recent report on burn-out in Flanders where 21% of teachers reported burn-out symptoms in 2019. This is higher than in the general Flemish population with 13% of individuals reporting burn-out symptoms (17). Previous research indeed indicated that teachers have more burn-out symptoms compared to the general population (15, 16). A recent Belgian report by the Sociaal-Economische Raad van Vlaanderen (17) also highlighted that in 2019 mainly individuals in care and education reported more burn-out symptoms. Moreover, our results showed that the percentages of teachers with a high risk of burn-out syndrome and high need for recovery follow an almost identical pattern over time. This may be worrying, as a high risk of burn-out syndrome is more alarming when also recovery need is high (23).

Overall, the first lockdown had a positive effect on teachers' wellbeing. In the initial stages of the COVID-19 pandemic, significant decreases in risk of burn-out syndrome, emotional exhaustion, depersonalization and recovery need were found. During the first lockdown (i.e., Mar/Apr 2020), schools were closed and teachers did not have any educational tasks. This is also in line with the study by Hilger et al. (38) who found a decrease in fatigue and the demanding aspects of the teaching job. It should be mentioned that these results are based on measurements conducted in May 2020 and took place in Germany, where the COVID-19 measures differed slightly from those in Belgium.

However, after the first lockdown, increases in risk of burn-out syndrome, emotional exhaustion, and need for recovery were observed. This may be due to the fact that teachers had to adapt to a new way of teaching (i.e., hybrid teaching). Furthermore, they were putting their own health and the health of their students at risk. Moreover, due to the lockdown measures they were not able to lean on their social network to decompress. These findings are in line with other research demonstrating an increase in anxiety, burn-out syndrome and a decrease in general quality of life when teachers had to return to the classroom while the pandemic was still ongoing (10, 11).

Despite these increases, summer vacations had a clear positive impact on teachers' risk of burn-out syndrome and recovery need. Both during the summer holiday of 2020 and 2021, a clear decrease in risk of burn-out syndrome and need for recovery was visible. During these months, measures were less strict and teachers did not have to teach. Nevertheless, burn-out syndrome level and recovery need seemed to be higher during the second summer holidays (2021), showing a possible negative long-term impact of the COVID-19 pandemic (summer holidays 2020 vs. 2021: risk of burn-out syndrome: 14.6 vs. 20.9%; recovery need: 34.0 vs. 39.8%). Moreover, the percentage of teachers having risk of burn-out syndrome during the summer holidays 2021 (i.e., 20.9%) was at the same level as the percentage in Sep/Oct 2019 (i.e., 20.8%). These findings suggest that teachers suffered from an additional mental burden due to the lockdown measures which disabled them to recover properly during the summer recess. In contrast to the other burn-out syndrome dimensions, a negative impact of the summer vacation on personal accomplishment was found, as a significant decrease at both time points (i.e., summer vacation 2020 and 2021) was observed. We reason that this might be related to the prospect of going back to teaching face-to-face while the pandemic was still going on. Moreover, when teachers are not teaching and thus decompressing, they might start to self-reflect and doubt themselves opposed to when they get immediate validation while they are teaching. These findings are in line with other research showing teachers to have high levels of stress and anxiety about the anticipation of schools reopening (10, 40, 50).

The first time point of school year 2020–2021 (i.e., T6; Jan/Feb 2021) again showed significant increases in risk of burn-out syndrome, emotional exhaustion, depersonalization and recovery need. It was shown in the general population that wellbeing and mental health decrease when lockdowns go on for longer periods of time (25). Our findings clearly indicate that there was a negative long-term impact of the COVID-19 pandemic on teachers' mental health.

An important strength of the present cohort study is its prospective time series design enabling to assess the effect of the COVID-19 pandemic on risk of burn-out syndrome, its dimensions and recovery need in secondary school teachers. These natural experiment data are unique and give a lot more information compared to any (retrospective) cross-sectional studies. Moreover, as the installation of a control group during such a pandemic is not possible, we aimed to reduce bias by installing several (control) measurement points over time. It should be mentioned though, that without a control group, it is still difficult to unravel causal effects of the pandemic from natural fluctuations throughout the school year.

A first limitation to this study is the fact that no data were collected during September–October 2020 and November–December 2020, as the final moment of data collection of the initial study was planned for August 2020. Although not intended, we decided (on an *ad hoc* basis) to prolong the initial study protocol and monitor the long-term effects of the pandemic. As the school year was already up and running, we were only able to monitor from January onwards.

Second, selection bias is likely to be present. Teachers were recruited on a voluntary basis, and thus teachers having high risk of burn-out syndrome might have been less willing to participate in this study. This may have resulted in an underestimation of the prevalence of risk of burn-out syndrome and recovery need. Although we tried to address this issue by sampling participants from all secondary schools in Flanders, our study sample consisted of more females and less teachers between 20 and 29 years old compared to the teaching population in secondary education in Flanders. On the one hand, this may have resulted in a higher prevalence of risk of burn-out syndrome, as females are more susceptible for burn-out syndrome (51). On the other hand, a meta-analysis showed a small and negative correlation between age and burn-out syndrome, possibly resulting in a small underestimation of risk of burn-out syndrome (52). Nevertheless, this small underestimation may have been canceled out as our sample also consisted of more teachers between 30 and 39 years old compared to the teaching population in Flanders. Although generalizability may be compromised, it is difficult to predict how (i.e., in which direction) our results were affected by the observed selection bias. Similarly, the changes over time of risk of burn-out syndrome, its dimensions and recovery need may have been affected by the fact that the proportions of the sample regarding sex and age changed over time. Our drop-out analyses showed that, at each time point, the retention group consisted of more female as well as older teachers compared to the drop-out group. Again, both proportional changes in sex and age may have influenced the results (probably in the opposite direction) as they are both associated with burn-out (51, 52).

Third, factors such as technostress (i.e., fear of using technology), COVID-19 fear and seasonal conditions may also have had an impact on mental wellbeing, and thus the changes in burn-out syndrome risk and recovery need may not be solely caused by the pandemic. Research suggested that more technostress may cause higher levels of emotional exhaustion and lower levels of personal accomplishment (53). Despite the fact that teachers had to use technology more often in the beginning of the pandemic, which may have resulted in more technostress, the current study found an initial positive effect of the pandemic on emotional exhaustion and no effect on personal accomplishment. Furthermore, multiple studies found a positive relationship between COVID-19 fear and burnout (54–56). Since teachers were among the first to return to work in person, it would have been interesting to take this factor into account. Moreover, we also found fluctuations of burn-out syndrome risk and recovery need over time even before the pandemic. It is likely that there are periods of higher levels of burn-out syndrome risk and recovery need during a normal school year, possibly influenced by the weather and seasonal conditions. Previous research showed the weather to have an impact on depression (57), job satisfaction and wellbeing (58), while seasonal conditions may influence anxiety (59) and depression (59, 60).

Fourth, since COVID-19 measures were different among countries, and as our study only included secondary school teachers, our findings may not be generalized to other countries, nor teaching populations.

Finally, self-report questionnaires were used to measure risk of burn-out syndrome (i.e., UBOS-L) and recovery need (i.e., SIMPH), possibly resulting in social desirability bias. However, we do not expect this to have influenced the results as this way of measuring was applied systematically across all time points.

## Conclusions

During the initial stages of the pandemic, positive lockdown effects were visible; a lower percentage of teachers were at risk for burn-out syndrome, decreases in emotional exhaustion and depersonalization were found and less teachers showed a high need for recovery. However, in the long-term, negative effects became visible, as increases in risk of burn-out syndrome, emotional exhaustion, depersonalization and recovery need were observed. Although summer vacations should help to reduce the risk of burn-out syndrome and need for recovery, burn-out levels and recovery need seemed to be higher during the second summer holidays (2021) compared to the first one (2020), suggesting elevated mental burden due to the ongoing pandemic and related lockdown measures. This study highlights once again the importance for interventions to reduce secondary school teachers' risk of burn-out syndrome and recovery need, especially in such difficult pandemic times. We advise policy makers and schools to focus on developing tools and interventions that cushion the impact of the pandemic on mental wellbeing in teachers. Moreover, the current teacher training course should be adapted to include tools on how to teach online, while practicing teachers should be offered training courses on this topic. This will allow teachers to feel more comfortable to teach online in case of school closures. Lastly, it would benefit teachers to gain the necessary know-how on how to deal with stress and to keep their recovery need low.

## Data availability statement

The raw data supporting the conclusions of this article will be made available by the authors, without undue reservation.

## Ethics statement

The studies involving human participants were reviewed and approved by Medical Ethics Committee of the University Hospital Brussels. The patients/participants provided their written informed consent to participate in this study.

## Author contributions

HDL, YV, EVH, and TD wrote and revised this manuscript. KDM and EZ reviewed this manuscript. YV collected the data for this manuscript. All authors contributed to the article and approved the submitted version.
