# Supplementary material for: Impact of the COVID-19 pandemic on risk of burn-out syndrome and recovery need among secondary school teachers in Flanders: A prospective study
Source: Front Public Health. 2022 Dec 12;10:1046435. doi: 10.3389/fpubh.2022.1046435 (PMC9792144; doi:10.3389/fpubh.2022.1046435)
Supplement: Supplementary file 1 [file Data_Sheet_1.docx]

**S1 APPENDIX: MODEL SELECTION PROCEDURE**

**MODEL SELECTION – RISK OF BURN-OUT SYNDROME**

A generalized linear mixed model was constructed with risk of burn-out syndrome as outcome and time as a predictor variable. The first level predictors gender and age were added to the model. Age significantly impacted the outcome variable and gender marginally impacted the outcome variable. Since gender was only marginally significant, it was further explored whether it should be retained in the model by comparing the AIC of both models and performing an ANOVA between the model with and without gender. The model with age and without gender (AIC = 4779.8) was preferred over the model with age and gender (AIC = 4791.6). ANOVA comparison between both models revealed no significant difference (chi² = 0.0, p = 1.0). The reduced model with age as first level predictor was retained. The second level predictor teaching hours was added to the model. Teaching hours did not significantly impact the outcome variable however this model (AIC = 4518.5) was still preferred over the model without teaching hours (AIC = 4779.8).

**MODEL SELECTION – EMOTIONAL EXHAUSTION**

A general linear mixed model was constructed with emotional exhaustion as outcome and time as a predictor variable. The first level predictors gender and age were added to the model. Gender significantly impacted the outcome variable while age did not. The model without age but with gender (AIC = 15238.15) was preferred over the model with both age and gender (AIC = 15250.2). ANOVA comparison between the model with both gender and age and with only gender revealed no significant difference (chi² = 0.0041, p = 0.95). The reduced model with gender as first level predictor was retained. The second level predictor teaching hours was added to the model. Teaching hours did not significantly impact the outcome variable however this model (AIC =14342.88) was still preferred over the model without teaching hours (AIC = 15238.15).

**MODEL SELECTION – DEPERSONALISATION**

A general linear mixed model was constructed with depersonalisation as outcome and time as a predictor variable. The first level predictors gender and age were added to the model. Gender significantly impacted the outcome variable and age was marginally significant. Age was further explored to see whether it should be retained in the model. The model without age but with gender (AIC = 10633.53) had lower AIC over the model with age and gender (AIC = 10640.94). However, ANOVA comparison between the model with both gender and age and with only gender revealed a significant difference (chi² = 5.43, p = 0.02). The full model with gender and age as first level predictors was retained because the ANOVA analysis revealed a significant difference between the two. The second level predictor teaching hours was added to the model. Teaching hours did not significantly impact the outcome variable however the model with teaching hours (AIC = 10086.07) was still preferred over the model without teaching hours (AIC = 10633.53).

**MODEL SELECTION – PERSONAL ACCOMPLISHMENT**

A general linear mixed model was constructed with personal accomplishment as outcome and time as a predictor variable. The first level predictors gender and age were added to the model. Both age and gender significantly impacted the outcome. The second level predictor teaching hours was added to the model. Teaching hours did not significantly impact the outcome variable however, this model (AIC = 11455.55) was still preferred over the model without teaching hours (AIC = 12211.08).
