# Supplementary material for: Impact of the COVID-19 pandemic on risk of burn-out syndrome and recovery need among secondary school teachers in Flanders: A prospective study
Source: Front Public Health. 2022 Dec 12;10:1046435. doi: 10.3389/fpubh.2022.1046435 (PMC9792144; doi:10.3389/fpubh.2022.1046435)
Supplement: Supplementary file 2 [file Data_Sheet_2.docx]

**S2 APPENDIX: REPRESENTATIVENSS OF THE BASELINE SAMPLE (T0)**

**Table A1. Representativeness of the baseline sample (T0)**

|  | **n** |  | **Chi²** | **p-value** | |
| --- | --- | --- | --- | --- | --- |
|  | **Sample**  **(n = 2,167)** | **Population**  **(n = 77,802)** |  |  |  |
| **Sex (n (%))**  Males  Females | 486 (22.4)  1,681 (77.6) | 27,300 (35.1)  50,502 (64.9) | 149.07  149.07 | **<0.001**  **<0.001** |  |
|  | **Sample**  **(n = 2,167)** | **Population**  **(n = 77,802)** |  |  |  |
| **Age (years) (n (%))**  20-29  30-39  40-49  50-59  +60 | 265 (12.2)  705 (32.5)  615 (28.4)  475 (21.9)  107 (4.9) | 11,508 (14.8)  22,844 (29.4)  20,876 (26.8)  18,071 (23.2)  4,503 (5.8) | 11.03  10.21  2.57  2.02  2.80 | **<0.001**  **0.001**  0.108  0.155  0.094 |  |
|  | **Sample**  **(n = 1,698)** | **Population**  **(n = 77,802)** |  |  |  |
| **Education network**  **(n (%))**  Flemish community schools  Subsidised free schools  Subsidised public schools | 871 (51.3)  768 (45.2)  59 (3.5) | 17,471 (22.5)  52,937 (68.0)  7,349 (9.4) | 778.72  394.49  70.12 | **<0.001**  **<0.001**  **<0.001** |  |

**Table A2. Drop-out analyses between each consecutive time point**

|  | **T0-T1** | | **T1-T2** | | **T2-T3** | | **T3-T4** | | **T4-T5** | | **T5-T6** | |
| --- | --- | --- | --- | --- | --- | --- | --- | --- | --- | --- | --- | --- |
|  | **C²/*t*** | ***p*** | **C²/*t*** | ***p*** | **C²/*t*** | ***p*** | **C²/*t*** | ***p*** | **C²/*t*** | ***p*** | **C²/*t*** | ***p*** |
| **Risk of Burn-out syndrome** | 0.184 | 0.668 | 0.350 | 0.554 | 0.041 | 0.839 | 0.577^-6^ | 0.999 | 0.240^-29^ | 1 | 0.122^-28^ | 1 |
| **Recovery need** | 0.941 | 0.347 | 2.011 | 0.0448* | 1.067 | 0.287 | 1.101 | 0.272 | 0.940 | 0.348 | 0.712 | 0.477 |
| **Emotional exhaustion** | -0.656 | 0.512 | 1.825 | 0.0685 | 0.013 | 0.989 | 1.543 | 0.124 | 0.747 | 0.456 | 0.716 | 0.475 |
| **Depersonalisation** | 0.995 | 0.320 | -0.107 | 0.915 | 0.228 | 0.820 | 1.452 | 0.148 | -0.485 | 0.628 | 1.411 | 0.160 |
| **Personal accomplishment** | -0.839 | 0.401 | 0.416 | 0.677 | -1.213 | 0.226 | -0.805 | 0.422 | 0.838 | 0.403 | -1.510 | 0.133 |

|  | **T6-T7** | | **T7-T8** | | **T8-T9** | |
| --- | --- | --- | --- | --- | --- | --- |
|  | **C²/*t*** | ***p*** | **C²/*t*** | ***p*** | **C²/*t*** | ***p*** |
| **Risk of Burn-out syndrome** | 0.229 | 0.633 | 0.019 | 0.891 | 0.452 | 0.501 |
| **Recovery need** | 0.545 | 0.586 | 1.687 | 0.093** | -0.550 | 0.583 |
| **Emotional exhaustion** | 0.508 | 0.612 | 1.479 | 0.140 | 0.235 | 0.814 |
| **Depersonalisation** | -0.754 | 0.451 | 0.589 | 0.556 | 0.440 | 0.660 |
| **Personal accomplishment** | 1.083 | 0.279 | 0.044 | 0.965 | 0.918 | 0.359 |

*Note. *p* < .05, ** *p* < .01, ****p* < .001

**Table A3. Drop-out analyses between each time point and baseline (T0)**

|  | **T0-T1** | | **T0-T2** | | **T0-T3** | | | **T0-T4** | | | **T0-T5** | | |
| --- | --- | --- | --- | --- | --- | --- | --- | --- | --- | --- | --- | --- | --- |
|  | **C²/*t*** | ***p*** | **C²/*t*** | ***p*** | **C²/*t*** | ***p*** | **C²/*t*** | | ***p*** | **C²/*t*** | | ***p*** |  |
| **Risk of Burn-out syndrome** | 0.184 | 0.668 | 1.478 | 0.224 | 0.173 | 0.677 | 0.010 | | 0.920 | 0.608^-28^ | | 1 |  |
| **Recovery need** | 0.941 | 0.347 | 1.069 | 0.285 | 1.176 | 0.240 | 1.345 | | 0.179 | 1.375 | | 0.170 |  |
| **Emotional exhaustion** | -0.656 | 0.512 | 0.808 | 0.419 | 0.620 | 0.536 | 0.490 | | 0.625 | 0.094 | | 0.925 |  |
| **Depersonalisation** | 0.995 | 0.320 | -0.181 | 0.857 | 0.940 | 0.347 | 0.238 | | 0.812 | 0.826 | | 0.409 |  |
| **Personal accomplishment** | -0.839 | 0.401 | -0.211 | 0.832 | 0.153 | 0.878 | 0.344 | | 0.731 | -0.321 | | 0.748 |  |
| **Age** | 4.290 | < 0.001*** | 6.346 | < 0.001*** | 8.302 | < 0.001*** | 6.612 | | < 0.001*** | 6.922 | | < 0.001*** |  |
| **Sex** | 0.328 | 0.567 | 1.249 | 0.264 | 2.720 | 0.099 | 0.959 | | 0.327 | 1.694 | | 0.193 |  |

|  | **T0-T6** | | | **T0-T7** | | | **T0-T8** | | | **T0-T9** | |
| --- | --- | --- | --- | --- | --- | --- | --- | --- | --- | --- | --- |
|  | **C²/*t*** | ***p*** | **C²/*t*** | | ***p*** | **C²/*t*** | | ***p*** | **C²/*t*** | | ***p*** |
| **Risk of Burn-out syndrome** | 0.250 | 0.617 | 0.052 | | 0.820 | 0.552 | | 0.458 | 0.035 | | 0.853 |
| **Recovery need** | 0.602 | 0.548 | 1.200 | | 0.230 | 1.359 | | 0.175 | -0.014 | | 0.989 |
| **Emotional exhaustion** | -1.139 | 0.255 | -0.666 | | 0.506 | -0.054 | | 0.957 | -0.229 | | 0.819 |
| **Depersonalisation** | 1.607 | 0.108 | 1.045 | | 0.297 | 1.431 | | 0.153 | 0.769 | | 0.442 |
| **Personal accomplishment** | -0.695 | 0.487 | -0.441 | | 0.660 | 0.017 | | 0.987 | -0.547 | | 0.585 |
| **Age** | 7.070 | < 0.001*** | 6.728 | | < 0.001*** | 7.196 | | < 0.001*** | 6.071 | | < 0.001*** |
| **Sex** | 7.709 | < 0.05* | 8.630 | | < 0.05* | 7.267 | | < 0.05* | 4.502 | | < 0.05* |

*Note. *p* < .05, ** *p* < .01, ****p* < .001
