# Supplementary material for: Impact of the COVID-19 pandemic on risk of burn-out syndrome and recovery need among secondary school teachers in Flanders: A prospective study
Source: Front Public Health. 2022 Dec 12;10:1046435. doi: 10.3389/fpubh.2022.1046435 (PMC9792144; doi:10.3389/fpubh.2022.1046435)
Supplement: Supplementary file 3 [file Data_Sheet_3.docx]

**S3 APPENDIX: Percentages of risk of burn-out syndrome and recovery need across all time points**

**Table B1. Risk of Burn-out syndrome and recovery need at each time point (%)**

|  | **Sept/Oct 2019** | **Nov/Dec 2019** | **Jan/Feb 2020** | **Mar/Apr2020** | **May/Jun 2020** | **Jul/Aug 2020** | **Jan/Feb 2021** |
| --- | --- | --- | --- | --- | --- | --- | --- |
| **Risk of Burn-out syndrome (%)** | 20.8 | 26.2* | 25.0 | 15.7* | 20.9* | 14.6* | 28.1* |
| **Recovery need (%)** | 55.7 | 57.4* | 55.8 | 38.2* | 46.9* | 34.0* | 61.3* |

|  | **Mar/Apr 2021** | **May/Jun 2021** | **Jul/Aug 2021** |
| --- | --- | --- | --- |
| **Risk of Burn-out syndrome (%)** | 26.3 | 30.8* | 20.9* |
| **Recovery need (%)** | 61.4 | 57.8 | 39.8* |

*Time point significantly different (p<0.05) from previous time point (statistical inferences are based on the calculated odds ratios and their confidence intervals)
