# Supplementary material for: Impact of the COVID-19 pandemic on risk of burn-out syndrome and recovery need among secondary school teachers in Flanders: A prospective study
Source: Front Public Health. 2022 Dec 12;10:1046435. doi: 10.3389/fpubh.2022.1046435 (PMC9792144; doi:10.3389/fpubh.2022.1046435)
Supplement: Supplementary file 4 [file Data_Sheet_4.docx]

**S4 APPENDIX: ESTIMATED MARGINAL MEANS/PROBABILITIES AND STANDARD ERRORS ACROSS ALL TIME POINTS**

**Table C1. Emotional exhaustion, depersonalisation, personal accomplishment, recovery need and risk of burn-out syndrome at each time point (Estimated marginal means/probabilities ± SE)**

|  | **Sept/Oct 2019** | **Nov/Dec 2019** | **Jan/Feb 2020** | **Mar/Apr2020** | **May/Jun 2020** | **Jul/Aug 2020** |
| --- | --- | --- | --- | --- | --- | --- |
|  | **EMM (SE)** | **EMM (SE)** | **EMM (SE)** | **EMM (SE)** | **EMM (SE)** | **EMM (SE)** |
| **Emotional exhaustion**  **Depersonalisation**  **Personal accomplishment** | 2.30(0.03)  1.18(0.02)  4.08(0.03) | 2.40(0.04)*  1.33(0.03)*  3.88(0.03)* | 2.37(0.04)  1.34(0.03)  3.96(0.03)* | 1.86(0.04)*  1.21(0.03)*  3.94(0.04) | 2.07(0.05)*  1.20(0.03)  3.95(0.04) | 1.84(0.04)*  1.22(0.04)  3.87(0.04)* |
| **Risk of Burn-out syndrome** | 0.025(0.008) | 0.042(0.014)* | 0.047(0.016) | 0.014(0.005)* | 0.026(0.010)* | 0.011(0.005)* |
| **Recovery need** | 2.54(0.05) | 2.68(0.06)* | 2.65(0.07) | 1.85(0.07)* | 2.21(0.08)* | 1.73(0.08)* |

*Time point significantly different (p<0.05) from previous time point

|  | **Jan/Feb 2021** | **Mar/Apr 2021** | **May/Jun 2021** | **Jul/Aug 2021** |  |
| --- | --- | --- | --- | --- | --- |
|  | **EMM (SE)** | **EMM (SE)** | **EMM (SE)** | **EMM (SE)** |  |
| **Emotional exhaustion**  **Depersonalisation**  **Personal accomplishment** | 2.49(0.04)*  1.36(0.03)*  3.87(0.04) | 2.55(0.05)  1.35(0.04)  3.92(0.04) | 2.52(0.05)  1.44(0.04)*  3.95(0.06) | 1.93(0.05)*  1.32(0.04)*  3.80(0.04)* |  |
| **Risk of Burn-out syndrome** | 0.058(0.020)* | 0.051(0.019) | 0.087(0.031)* | 0.032(0.013)* |  |
| **Recovery need** | 2.83(0.0)8* | 2.97(0.09) | 2.87(0.10) | 1.75(0.09)* |  |

*Time point significantly different (p<0.05) from previous time point

| **Table C2. Odds ratios of the predictors of risk of burn-out syndrome** | | | |
| --- | --- | --- | --- |
|  | **Risk of Burn-out syndrome** | | |
| *Predictors* | *Odds Ratios* | *CI* | *p* |
| Sep/Oct 2019 – Nov/Dec 2019 | 1.83 | 1.34 – 2.49 | **<0.001** |
| Nov/Dec 2019 – Jan/Feb 2020 | 0.88 | 0.62 – 1.27 | 0.499 |
| Jan/Feb 2020 – Mar/Apr 2020 | 0.33 | 0.21 – 0.51 | **<0.001** |
| Mar/Apr 2020 – May/Jun 2020 | 2.61 | 1.60 – 4.26 | **<0.001** |
| May/Jun 2020 – Jul/Aug 2020 | 0.32 | 0.19 – 0.55 | **<0.001** |
| Jul/Aug 2020 – Jan/Feb 2021 | 5.30 | 3.09 – 9.09 | **<0.001** |
| Jan/Feb 2021 – Mar/Apr 2021 | 0.93 | 0.56 – 1.54 | 0.765 |
| Mar/Apr 2021 – May/Jun 2021 | 1.85 | 1.04 – 3.27 | **0.036** |
| May/Jun 2021 – Jul/Aug 2021 | 0.28 | 0.15 – 0.51 | **<0.001** |
| Age | 0.99 | 0.99 – 0.99 | **<0.001** |
| Teaching hours per week | 1.00 | 1.00 – 1.00 | 0.839 |

Model adjusted for age and teaching hours per week

*CI = confidence interval*

*Significant p-values are marked in bold*

| **Table C3. Estimates of the predictors for emotional exhaustion** | | | |  |  |
| --- | --- | --- | --- | --- | --- |
| **Predictors** | **Emotional exhaustion** | | |  |  |
|  | **Estimates** | **CI** | ***p*** | ***t*** | ***d*** |
| Sep/Oct 2019 – Nov/Dec 2019 | 0.11 | 0.05 – 0.18 | **0.001** | 3.398 | 0.162 |
| Nov/Dec 2019 – Jan/Feb 2020 | -0.04 | -0.12 – 0.03 | 0.280 | -1.081 | 0.061 |
| Jan/Feb 2020 – Mar/Apr 2020 | -0.51 | -0.60 – -0.43 | **< 0.001** | -11.724 | 0.728 |
| Mar/Apr 2020 – May/Jun 2020 | 0.22 | 0.12 – 0.32 | **< 0.001** | 4.442 | 0.312 |
| May/Jun 2020 – Jul/Aug 2020 | -0.22 | -0.32 – -0.11 | **< 0.001** | -4.088 | 0.312 |
| Jul/Aug 2020 – Jan/Feb 2021 | 0.66 | 0.56 – 0.76 | **< 0.001** | 12.387 | 0.938 |
| Jan/Feb 2021 – Mar/Apr 2021 | 0.03 | -0.08 – 0.14 | 0.582 | 0.550 | 0.044 |
| Mar/Apr 2021 – May/Jun 2021 | 0.02 | -0.10 – 0.15 | 0.738 | 0.334 | 0.030 |
| May/Jun 2021 – Jul/Aug 2021 | -0.63 | -0.75 – -0.50 | **< 0.001** | -9.548 | 0.889 |
| Sex (ref. category = male) | 0.24 | 0.11 – 0.36 | **< 0.001** | 3.691 |  |
| Teaching hours per week | 0.00 | -0.00 – 0.00 | 0.574 | 0.563 |  |

Model adjusted for sex and teaching hours per week

*CI = confidence interval*

*Significant p-values are marked in bold*

| **Table C4. Estimates of the predictors for depersonalisation** | | | |  |  |
| --- | --- | --- | --- | --- | --- |
|  | **Depersonalisation** | | |  |  |
| *Predictors* | *Estimates* | *CI* | *p* | *t* | *d* |
| Sep/Oct 2019 – Nov/Dec 2019 | 0.15 | 0.10 – 0.19 | **<0.001** | 6.582 | 0.315 |
| Nov/Dec 2019 – Jan/Feb 2020 | 0.01 | -0.04 – 0.06 | 0.724 | 0.353 | 0.020 |
| Jan/Feb 2020 – Mar/Apr 2020 | -0.13 | -0.18 – -0.07 | **<0.001** | -4.307 | 0.268 |
| Mar/Apr 2020 – May/Jun 2020 | -0.01 | -0.08 – 0.05 | 0.718 | -0.362 | 0.025 |
| May/Jun 2020 – Jul/Aug 2020 | 0.02 | -0.05 – 0.09 | 0.571 | 0.567 | 0.043 |
| Jul/Aug 2020 – Jan/Feb 2021 | 0.13 | 0.06 – 0.20 | **<0.001** | 3.759 | 0.285 |
| Jan/Feb 2021 – Mar/Apr 2021 | -0.01 | -0.08 – 0.07 | 0.856 | -0.181 | 0.014 |
| Mar/Apr 2021 – May/Jun 2021 | 0.09 | 0.01 – 0.18 | **0.032** | 2.149 | 0.195 |
| May/Jun 2021 – Jul/Aug 2021 | -0.12 | -0.21 – -0.04 | **0.005** | -2.833 | 0.264 |
| Sex (ref. category = male) | -0.14 | -0.22 – -0.05 | **0.002** | -3.154 |  |
| Age | 0.00 | 0.00 – 0.01 | **0.024** |  |  |
| Teaching hours per week | 0.00 | -0.00 – 0.00 | 0.335 |  |  |

Model adjusted for sex, age, and teaching hours per week

*CI = confidence interval*

*Significant p-values are marked in bold*

| **Table C5. Estimates of the predictors for personal accomplishment** | | | |  |  |  |
| --- | --- | --- | --- | --- | --- | --- |
|  | **Personal accomplishment** | | |  |  |  |
| *Predictors* | *Estimates* | *CI* | *p* |  | *t* | *d* |
| Sep/Oct 2019 – Nov/Dec 2019 | -0.19 | -0.25 – -0.14 | **<0.001** |  | -7.469 | 0.356 |
| Nov/Dec 2019 – Jan/Feb 2020 | 0.08 | 0.02 – 0.14 | **0.009** |  | 2.619 | 0.147 |
| Jan/Feb 2020 – Mar/Apr 2020 | -0.03 | -0.09 – 0.04 | 0.410 |  | -0.824 | 0.051 |
| Mar/Apr 2020 – May/Jun 2020 | 0.02 | -0.06 – 0.09 | 0.673 |  | 0.421 | 0.030 |
| May/Jun 2020 – Jul/Aug 2020 | -0.08 | -0.16 – -0.00 | **0.048** |  | -1.976 | 0.151 |
| Jul/Aug 2020 – Jan/Feb 2021 | 0.00 | -0.08 – 0.08 | 0.932 |  | 0.085 | 0.006 |
| Jan/Feb 2021 – Mar/Apr 2021 | 0.04 | -0.04 – 0.13 | 0.321 |  | 0.992 | 0.079 |
| Mar/Apr 2021 – May/Jun 2021 | 0.04 | -0.06 – 0.13 | 0.463 |  | 0.773 | 0.066 |
| May/Jun 2021 – Jul/Aug 2021 | -0.16 | -0.26 – -0.06 | **0.002** |  | -3.073 | 0.286 |
| Sex (ref. category = male) | -0.14 | -0.23 – -0.05 | **0.003** |  | -2.985 |  |
| Age | 0.00 | 0.00 – 0.01 | **0.010** |  | 2.574 |  |
| Teaching hours per week | -0.00 | -0.00 – 0.00 | 0.642 |  | -0.465 |  |

Model adjusted for sex, age, and teaching hours per week

*CI = confidence interval*

*Significant p-values are marked in bold*

| **Table C6. Estimates of the predictors for recovery need** | | | |  |  |
| --- | --- | --- | --- | --- | --- |
|  | **Recovery need** | | |  |  |
| *Predictors* | *Estimates* | *CI* | *p* | *t* | *d* |
| Sep/Oct 2019 – Nov/Dec 2019 | 0.14 | 0.03 – 0.25 | **0.010** | 27.374 | 0.122 |
| Nov/Dec 2019 – Jan/Feb 2020 | -0.04 | -0.17 – 0.09 | 0.582 | 2.582 | 0.031 |
| Jan/Feb 2020 – Mar/Apr 2020 | -0.79 | -0.94 – -0.65 | **<0.001** | -0.550 | 0.667 |
| Mar/Apr 2020 – May/Jun 2020 | 0.36 | 0.19 – 0.52 | **<0.001** | -10.763 | 0.301 |
| May/Jun 2020 – Jul/Aug 2020 | -0.48 | -0.65 – -0.30 | **<0.001** | 4.292 | 0.402 |
| Jul/Aug 2020 – Jan/Feb 2021 | 1.10 | 0.93 – 1.28 | **<0.001** | -5.281 | 0.928 |
| Jan/Feb 2021 – Mar/Apr 2021 | 0.14 | -0.05 – 0.32 | 0.149 | 12.292 | 0.115 |
| Mar/Apr 2021 – May/Jun 2021 | -0.10 | -0.31 – 0.11 | 0.351 | 1.442 | 0.085 |
| May/Jun 2021 – Jul/Aug 2021 | -1.12 | -1.34 – -0.91 | **<0.001** | -0.934 | 0.947 |
| Sex (ref. category = male) | 0.44 | 0.27 – 0.62 | **<0.001** | 4.903 |  |
| Teaching hours per week | 0.00 | -0.00 – 0.00 | 0.741 | 0.331 |  |

Model adjusted for sex and teaching hours per week

*CI = confidence interval*

*Significant p-values are marked in bold*
